# Proteomic analysis of Toraya buffalo seminal plasma and sperm: uncovering insights to optimize reproductive success

**DOI:** 10.3389/fvets.2025.1492135

**Published:** 2025-04-10

**Authors:** Tulus Maulana, Syahruddin Said, Raden Iis Arifiantini, Jakaria Jakaria, Asep Gunawan

**Affiliations:** ^1^Graduate School of Animal Production and Technology, Faculty of Animal Science, IPB University, Bogor, Indonesia; ^2^Research Center for Applied Zoology, National Research and Innovation Agency, Bogor, Indonesia; ^3^Division of Veterinary Reproduction and Obstetrics, School of Veterinary Medicine and Biomedical Sciences, IPB University, Bogor, Indonesia; ^4^Department of Animal Production and Technology, Faculty of Animal Science, IPB University, Bogor, Indonesia

**Keywords:** proteome, seminal plasma, sperm, TCA cycle, Toraya buffalo

## Abstract

The characterization of sperm and seminal plasma proteins is essential for understanding bull fertility and optimizing reproductive success in buffalo bulls. Despite its importance, the reproductive proteomic of Toraya buffalo, an indigenous breed in Indonesia, remains largely unexplored. This study aimed to examine the seminal plasma and sperm proteins of Toraya buffalo to uncover those critical for reproductive functions. Semen samples were collected from eight Toraya buffalo bulls aged 4 to 10 years. Protein profiling was performed using one-dimensional sodium dodecyl sulfate-polyacrylamide gel electrophoresis (1D-SDS-PAGE), followed by in-gel digestion and liquid chromatography-tandem mass spectrometry (LC-MS/MS) analysis. Bioinformatics tools, including UniProt, PANTHER, DAVID, and STRING, were utilized to identify and annotate the detected proteins. This study successfully identified four key reproductive proteins: ADAM32 in seminal plasma and ZPBP, SPACA3, and CCDC136 in sperm. These proteins are essential for sperm motility, energy production, and acrosome formation, which are critical processes for fertilization. Additionally, many identified proteins were associated with metabolic pathways, particularly the tricarboxylic acid (TCA) cycle, which plays a fundamental role in energy supply for sperm function. In conclusion, this study offers the first comprehensive proteomic identification of seminal plasma and sperm proteins associated with reproductive functions in the Toraya buffalo. The findings highlight the presence of key proteins in sperm, including ZPBP, SPACA3, and CCDC136, as well as the identification of ADAM32 in seminal plasma, contributing to a deeper understanding of buffalo reproductive biology.

## 1 Introduction

The Toraya buffalo is an indigenous breed from Indonesia that plays a vital role in the agricultural and livestock systems of the region, particularly in Sulawesi (1). The breeding outcome of these buffaloes is essential for the efficiency and continuity of the livestock industry and local culture. In spite of their significance, issues concerning fertility and reproductive health are prevalent, underscoring the necessity for scientific methods to enhance breeding effectiveness and reproductive results.

Seminal plasma, a complex secretion from the testes, accessory sex glands, and epididymis, is essential for sperm fertility. It plays a critical role in sperm maturation, motility acquisition, capacitation, and the acrosome reaction (2). Sperm development is a complex process involving the proliferation and differentiation of male germ cells, with mature sperm undergoing final maturation and acquiring motility in the epididymis (3) Poor semen quality, particularly low sperm motility, is a primary indicator of this reproductive inefficiency and may be linked to an inadequate seminal microenvironment. Brohi et al. (4) reported, male infertility is a significant challenge, with underlying mechanisms that remain poorly understood.

Due to the essential function of proteins in these reproductive processes, comprehensive and systematic identification of sperm and seminal plasma proteins is vital for gaining new insights into sperm development, male fertility, and infertility. Proteomic analyses of sperm and seminal plasma have been conducted in various domestic animals, including buffalo (3, 5, 6), cattle (7–11), stallion (12) and sheep (13). To investigate the proteins involved in sperm development, we conducted an integrated proteomic study on buffalo sperm and seminal plasma. This approach enables the identification and characterization of key proteins that regulate sperm maturation, motility, and fertilization, offering insights into their roles in reproductive health. Understanding the protein profiles in sperm and seminal plasma is crucial, as variations can significantly impact fertility.

A comprehensive proteomic analysis by Gomes et al. (11) identified several key proteins in bovine seminal plasma, including BSPs (BSP1, BSP5, BSP3), spermadhesins, clusterin, osteopontin, and metalloproteinase inhibitor2. Similarly, Ashwita et al. (14) reported, proteomic profiling of spermatozoa and seminal plasma in *Bos indicus* bulls revealed proteins linked to semen quality, such as those involved in sperm protection, capacitation, and the acrosome reaction. These findings underscore the importance of identifying and characterizing proteins in sperm and seminal plasma, as their roles are critical to understanding and improving reproductive health and fertility outcomes. Despite their adaptability to hot and humid tropical climates, buffaloes exhibit low reproductive efficiency, a common issue among buffalo breeds (3).

By exploring the proteomic profiles of Toraya buffalo sperm and seminal plasma, this research represents a pioneering effort utilizing omics technology for the conservation of this important breed, which plays a crucial role in food security and local culture. The study aims to uncover valuable insights that can enhance reproductive biotechnology, optimize reproductive success, and characterize reproductive function proteins in the seminal plasma and sperm of Toraya buffalo through proteomic analysis.

## 2 Materials and methods

### 2.1 Experimental design and animal

The semen used in this study was collected from eight Toraya buffalo bulls, an endemic species native to Tanah Toraja Regency, South Sulawesi, Indonesia. The bulls were aged between four and ten years, with an average body weight of 400–600 kg. The experimental design and animal models used in this study were approved by the Animal Ethics Committee of the National Agency for Research and Innovation under certificate number 050/KE.02/SK/03/2023.

### 2.2 Semen collection and protein extraction

Semen samples were collected using an artificial vagina, a method that minimizes physiological alterations in the animals. The collection was conducted by experienced technicians to ensure proper handling and to maintain semen quality. Immediately after collection, semen quality was assessed through both macroscopic and microscopic evaluations. Only bulls with sperm motility exceeding 70% and a sperm concentration greater than 800 × 10⁶ cells/mL were included in this study, following the Indonesian National Standard (SNI) for fresh semen (15). Due to the exploratory nature of this study and time constraints, semen collection was performed only once. Toraya buffalo is a unique species indigenous to Toraja Regency, and semen collection required approval from the owners or local community leaders. These factors made repeated collection unfeasible, and thus, a single collection was performed for the purposes of this initial investigation.

### 2.3 Protein extraction and protein molecular weight analysis using 1D SDS-Page

The semen was then centrifuged at 6500 rpm, 4°C for 30 min to separate the sperm from the seminal plasma. The supernatant (seminal plasma) and the pellet (sperm) were carefully collected and stored at −20°C for subsequent analysis. The sperm pellets were extracted using PRO-PREPTM Protein Extraction Solution (iNtRON Biotechnology, Korea) according to the manufacturer’s instructions. A total of 400 μL of PRO-PREP solution was added to the sperm pellet and incubated at −20°C for 20 min. The mixture was then centrifuged at 13,000 rpm (4°C) for 5 min, and the supernatant was transferred into a sterile tube. The total soluble protein concentration of the seminal plasma and sperm samples was measured prior to SDS-PAGE analysis using the bicinchoninic acid (BCA) protein assay method (Thermo Scientific™, United States).

SDS-Page analysis was performed to determine the protein profile based on molecular weight, depicted in the form of bands on the gel. Protein separation was performed using SurePAGE™, Bis-Tris, 10 cm × 8 cm, 12 wells, 4–20% gradient gel (M00656; GenScript) (SurePAGE, GenScript Biotech Corp., Hong Kong), with running buffer Tris-MOPS-SDS (M00138; GenScript). Electrophoresis was performed at a voltage of 200 V and a current of 100–120 mA for 50 min. The gel was then stained using Coomassie Brilliant Blue stain (R-250; Bio-Rad, United States). The marker used was the Broad Multi Color Pre-Stained Protein Standard (M00624; GenScript) with a molecular weight range of ~5–270 kDa.

### 2.4 In gel tryptic digestion dan peptide clean up

The protein bands are excised into 1 mm × 1 mm pieces from an acrylamide gel and placed in sterile microcentrifuge tubes. Dye removal is achieved by adding 200 μL of a destaining solution (80 mg ammonium bicarbonate in 20 mL ACN and 20 mL ultrapure water) and incubating at 37°C for 30 min with gentle agitation, repeating the process once more. Cysteine groups are reduced by adding 30 μL of reduction solution (3.3 μL TCEP in 30 μL digestion buffer) and incubating at 60°C for 10 min. After discarding the reduction solution, alkylation is performed by adding 30 μL of alkylation solution (iodoacetamide in digestion buffer) and incubating for 1 h at room temperature in the dark. The gel pieces are then washed twice with 200 μL destaining solution at 37°C for 15 min. For digestion, 50 μL of ACN is added to dry the gel pieces for 15 min, followed by a 5–10 min air dry. Then, 10 μL of active trypsin solution (10 ng/μL) is added, incubated for 15 min, and then 25 μL digestion buffer is added for a 4 h incubation at 37°C or overnight at 30°C with agitation. The digestion is stopped by adding 10 μL of 1% TFA or formic acid and incubating for 5 min. Peptide purification is performed using Pierce C18 Spin Columns, first activating the resin with 200 μL of 50% ACN and equilibrating with 200 μL of 0.5% TFA in 5% ACN. A 150 μL sample is added, followed by centrifugation at 1500 g for 5 min to allow peptides to bind. The column is washed with 200 μL equilibration buffer and centrifuged, and peptides are eluted with 20 μL of 70% ACN. Eluted peptides are dried in a vacuum centrifuge (SpeedVac) and ready for LC–MS/MS analysis.

### 2.5 Peptide fractionation and LC–MS/MS analysis

The dried peptide sample was dissolved in 50 μL of a dissolving solution consisting of 2% ACN, 98% ultrapure water, and 0.1% formic acid. After that, the peptide sample was centrifuged at 12,000 rpm for 10 min. Next, 2.5 μL of the peptide was fractionated using the Nano LC Ultimate 3,000 Series System Tandem Q Exactive Plus Orbitrap HRMS (Thermo Scientific). The trap column used had a diameter of 30 μm and a length of 5 mm (Thermo Scientific™ 164,649), combined with a PepMap RSLC C18 capillary column (75 μm inner diameter X 15 cm, 3 μm particle size, 100 pore size, part number ES 800, Thermo Scientific) and a flow rate of 300 nL/min. The eluents used were H2O + 0.1% formic acid and Acetonitrile (A) + 0.1% formic acid (B). The elution process involved a gradient from 2 to 35% solvent B over 27 min, followed by a gradient from 35 to 99% solvent B over 10 min, then 99% solvent B for 15 min, and finally 2% solvent B for 30 min. Signal peptide was obtained using the LTQ-Orbitrap mass spectrometer (Thermo Scientific, Bremen, Germany) with a mass range of 200–2000 m/z.

### 2.6 Database analysis, protein classification, and bioinformatics

The data obtained from the LC–MS/MS instrument were analyzed using Proteome Discoverer 2.2 software with the Sequest HT search engine (16). The analysis was performed using the *Bos taurus* proteome database available at UniProt,^1^ applying false discovery rates of 1% (strict) and 5% (relaxed). The theoretical isoelectric point (pI) was calculated based on the amino acid sequences present in the database. The HT score, representing the sum of all Xcorr peptide values exceeding the defined threshold, was used for protein identification, Xcorr (cross-correlation) is a metric used to assess the alignment between experimentally derived peptide fragments and the theoretical spectrum, which consists of a series of b and y ions. Proteins were required to have an HT sequence score greater than 1 and at least two unique peptides, with a mass tolerance of 10 ppm. To further analyze the functions of the identified proteins, the PANTHER classification system^2^ and the Database for Annotation, Visualization, and Integrated Discovery (DAVID) v6.8^3^ were utilized. Venn analysis for group comparisons was conducted using the web-based tool available at https://bioinformatics.psb.ugent.be/webtools/Venn/. Additionally, protein–protein interactions were analyzed using STRING version 11.0.^4^

## 3 Results

### 3.1 Profiling and distribution of proteins in seminal plasma and sperm

**Figure 1:**
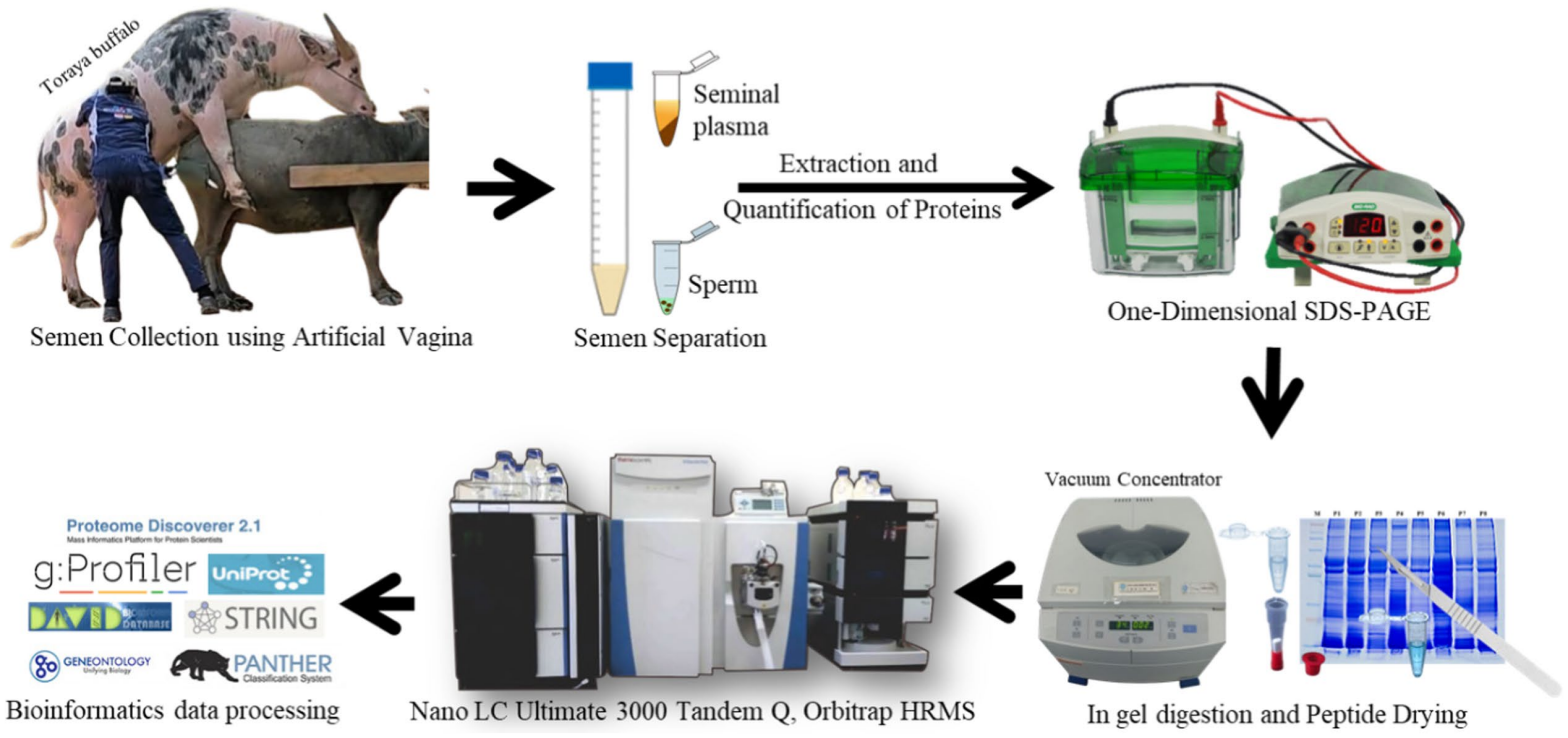
Scheme of proteomic analysis of Toraya buffalo seminal plasma and sperm.

The LC–MS/MS analysis, processed using Proteome Discoverer 2.2 software, identified a total of 1,893 proteins in seminal plasma and 1,913 proteins in sperm from eight Toraya buffalo bulls (Table 1). Protein identification in sperm and seminal plasma was based on the Xcorr value of peptides exceeding the predefined threshold (Sequest HT score). The most abundant proteins identified in sperm (Table 2) were TUBB4B (139.6), ATP5F1B (114.72), CCIN (72.62), RAB2A (67.07), and GAPDHS (69.27). In seminal plasma (Table 2), the five most abundant proteins were CLU (513.38), ALB (229.36), HSP90AA1 (161.9), A2M (127.57), and GPI (116.61) (Figure 1).

**Table 1:** Number of identified seminal plasma and sperm proteins of Toraya buffalo.

Protein	Toraya buffalo (*n* = 8)	
	Seminal plasma	Sperm
Proteome discover 2.2	1893	1913
Selection results	311	260

Proteome discover 2.2: expressed proteins from software analysis; Selection result: proteins that have been selected from protein duplication, unique protein ≥ 2, HT > 1.

**Table 2:** Summary of high abundant proteins in sperm and seminal plasma.

Protein name	Xcorr value	Gene name	Uniprot-ID	Molecular function
Sperm				
Tubulin beta-4B	139.96	TUBB4B	Q3MHM5	Major constituent of microtubules
ATP synthase subunit beta	114.72	ATP5F1B	A0A452DII8	Mitochondrial membrane ATP synthase
Calicin	72.62	CCIN	Q28068	Possible morphogenic cytoskeletal
GADPH	69.27	GAPDHS	A0A4W2CEF3	Energy production during spermatogenesis
Ras-related protein Rab-2A	67.07	RAB2A	A0A4W2I9W7	Vesicle transport leading to acrosome formation
Seminal plasma				
Clusterin	513.38	CLU	A0A4W2D277	Prevents aggregation of nonnative proteins
Albumin	229.36	ALB	A0A4W2D8T3	Main protein of plasma
HSP 90-alpha	161.9	HSP90AA1	A0A4W2G0N7	Key role in the cellular response to stress
Alpha-2-macroglobulin	127.57	A2M	Q7SIH1	Regulate immune responses
Glucose-6-phosphate isomerase	116.61	GPI	A0A4W2CDD2	Providing the energy

**Figure 2:**
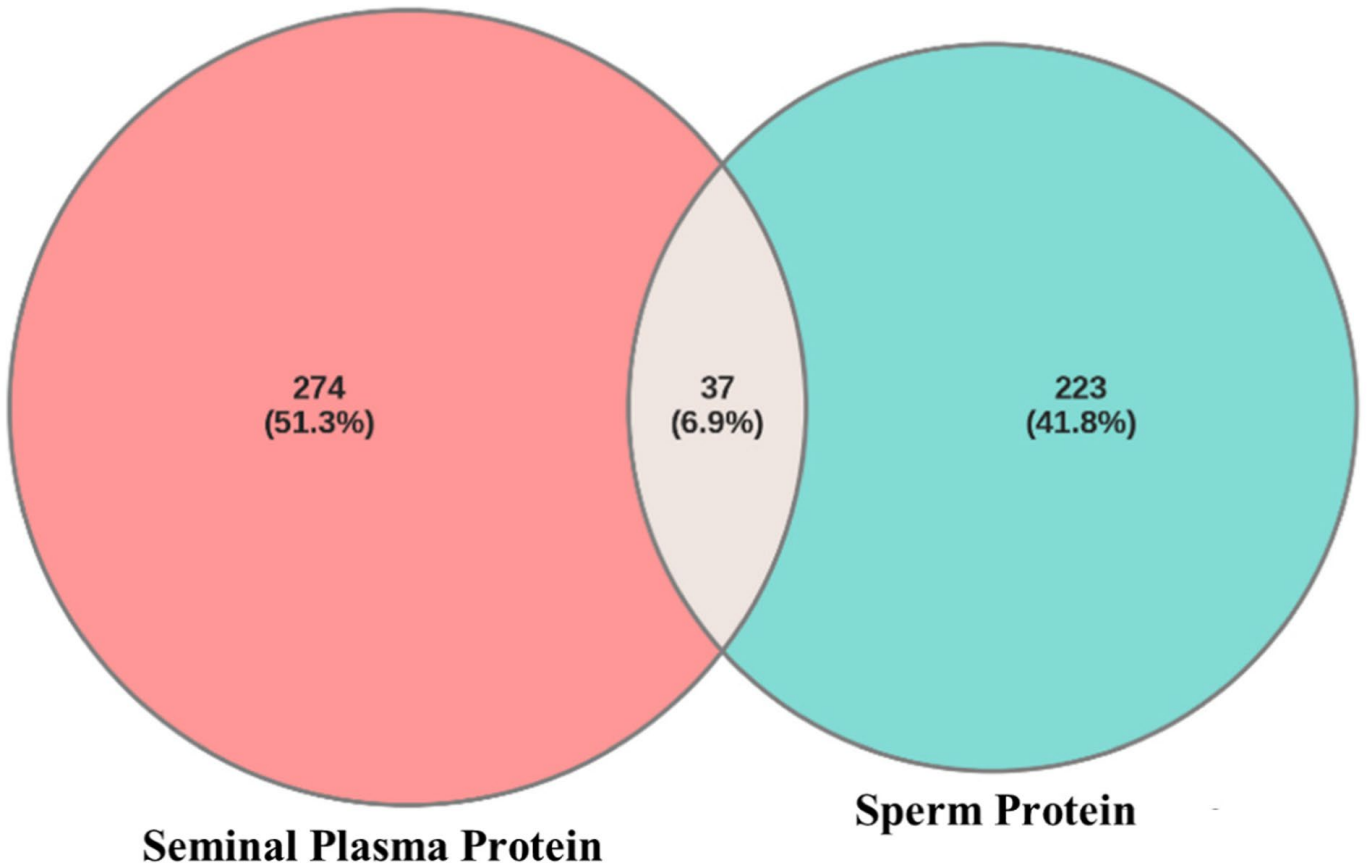
Venn diagram of proteins from Toraya buffalo seminal plasma and sperm.

Venn diagram analysis using Venny software (Figure 2) revealed that 37 proteins (6.9%) were expressed in both seminal plasma and sperm, while 274 proteins (51.3%) were uniquely expressed in seminal plasma and 223 proteins (41.8%) were uniquely expressed in sperm. The distribution of the proteome in seminal plasma and sperm of Toraya buffalo is illustrated in Figure 2, based on molecular weight (kDa), isoelectric point (pI), and the number of unique peptides. The highest proportion of proteins in both seminal plasma and sperm was observed within the molecular weight range of 41–60 kDa, while the lowest was detected at molecular weights ≤10 kDa and ≥ 200 kDa.

**Figure 3:**
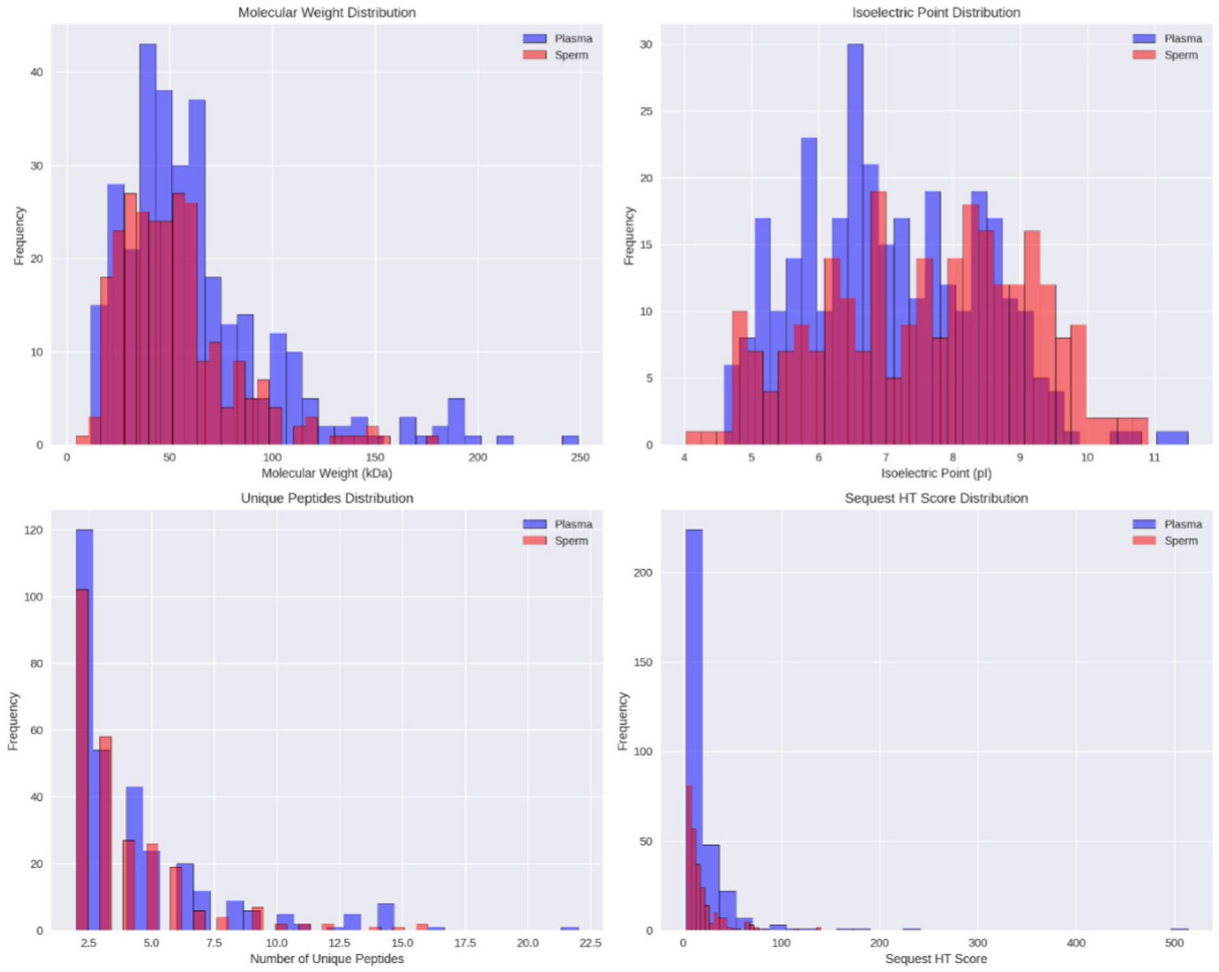
Distribution of identified proteins in Toraya buffalo sperm and seminal plasma. **(A)** molecular weight, **(B)** isoelectric point (pI), **(C)** number of unique peptides, **(D)** and sequest HT score.

When analyzed based on isoelectric point (pI), seminal plasma proteins were most abundant within the pI range of 6–7, with the lowest abundance at pI values ≥11. In contrast, sperm proteins were predominantly found within the pI range of 8–9, with the lowest abundance at pI values ≥11 (Figures 3A,B).

Regarding the number of unique peptides, the highest proportion was observed in proteins with a single unique peptide, comprising 275 proteins (46.93%) in seminal plasma and 260 proteins (50.10%) in sperm. Conversely, proteins with 10 unique peptides were the least represented, with only 5 proteins (0.85%) in seminal plasma and 2 proteins (0.39%) in sperm (Figures 3C,D). Additionally, several proteins containing more than 14 unique peptides were identified, including Tubulin beta-4B chain (Q3MHM5), ATP synthase subunit beta (A0A452DII8), and Calicin (Q28068) in sperm, as well as Alpha-2-macroglobulin (Q7SIH1), Cullin-associated NEDD8-dissociated protein (A0A6P5BT66), and Heat shock protein HSP 90-alpha (A0A4W2G0N7) in seminal plasma.

### 3.2 Gene ontology analysis of the proteins

**Figure 4:**
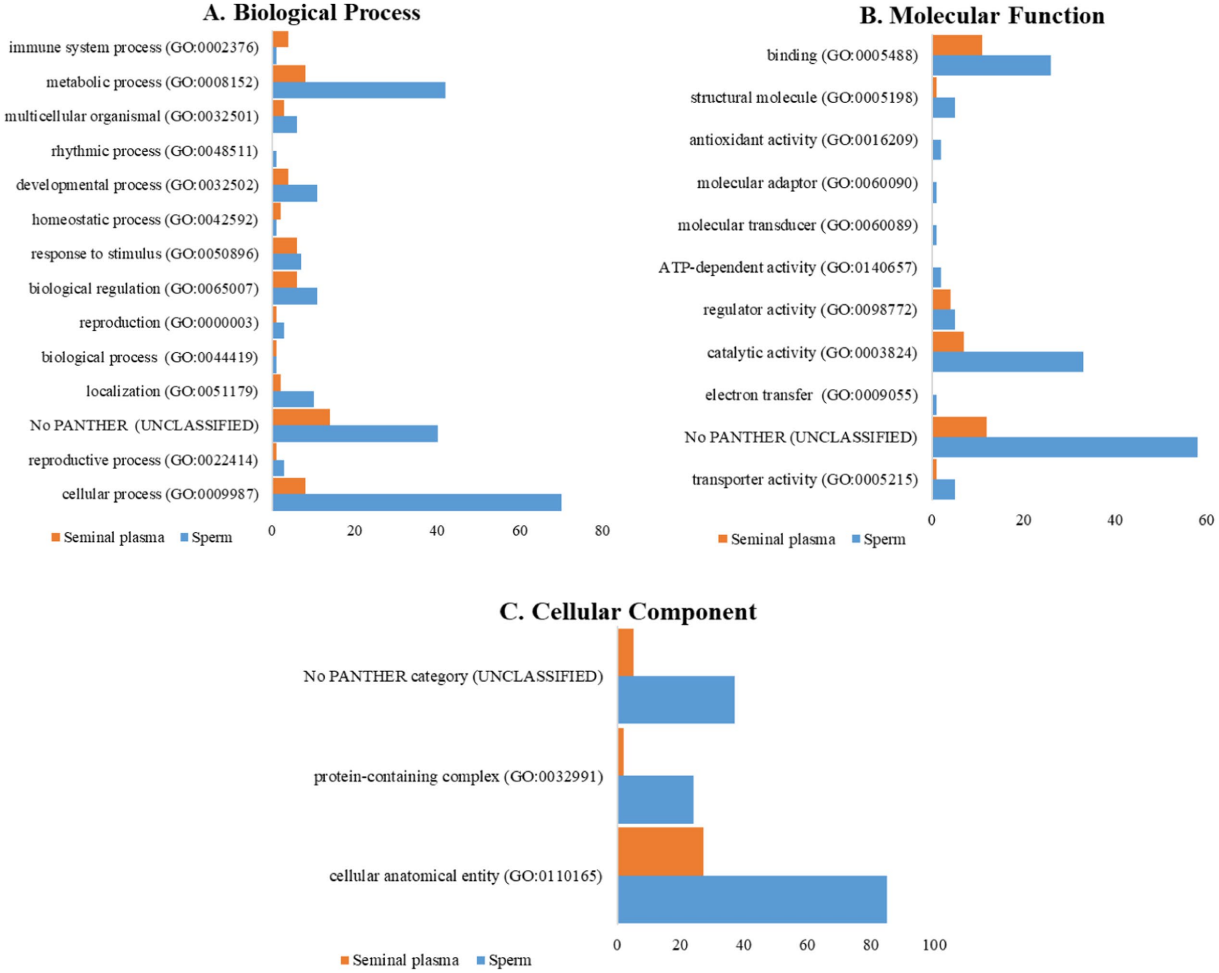
Comparison of proteins involved in **(A)** biological processes, **(B)** molecular functions, **(C)** and cellular components, in seminal plasma and sperm of Toraya buffalo.

The classification of proteins from PANTHER GO analysis of seminal plasma and sperm in Toraya buffalo revealed that there were proteins that remained unclassified or did not have any assigned category. This could be due to the limitations of the buffalo database, which still follows the *Bos taurus* database. Biological processes based on GO analysis revealed that cellular process (GO:0009987) and metabolic process (GO:0008152) were the most dominant in the seminal plasma and sperm of Toraya buffalo. The seminal plasma proteins were associated with reproduction (GO:0000003) and reproductive processes (GO:0022414), including ADAM32 (A0A3Q1LZ36), while the sperm proteins included ZPBP (F1N369), SPACA3 (A6QQ77), and CCDC136 (F1N343) (Figure 4A). The predominant molecular functions identified in seminal plasma were binding (GO:0005488) and catalytic activity (GO:0003824), as shown in Figure 4B. Additionally, the most dominant cellular component in both seminal plasma and sperm was the cellular anatomical entity (GO:0110165), as depicted in Figure 4C.

### 3.3 Seminal plasma and sperm protein interaction and pathway enrichment analysis

**Figure 5:**
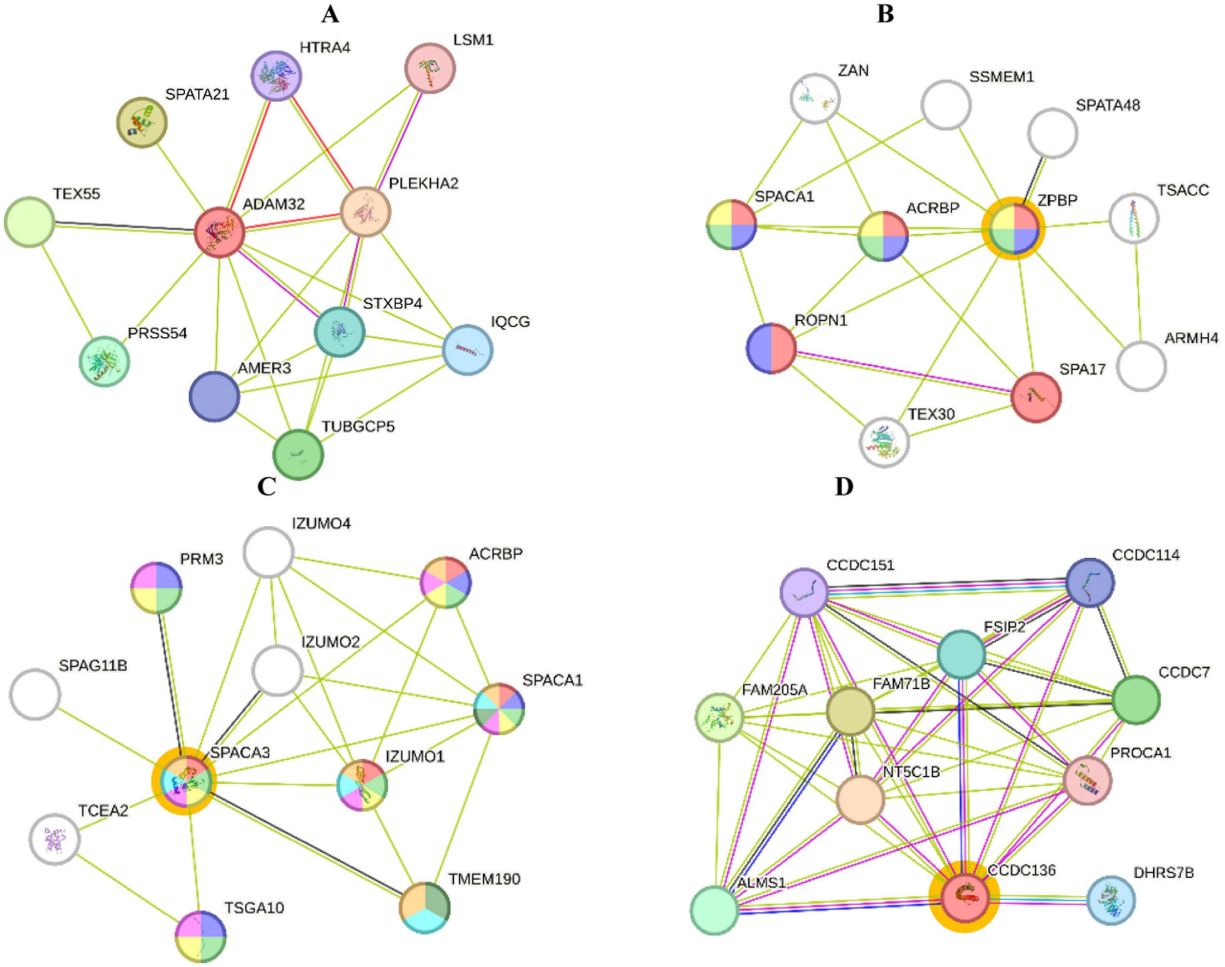
Protein interactions of Toraya buffalo. Seminal plasma protein **(A)** ADAM32, sperm protein, **(B)** ZPBP, **(C)** SPACA3, **(D)** and CCDC136 (STRING platform: http://string-db.org).

STRING analysis of expressed proteins related to reproductive function (GO:0000003) and reproductive process (GO:0022414) in seminal plasma (Figure 5A) showed that ADAM32 protein interacted with HTRA4, PLEKHA2, STXBP4, TUBGCP5, IQCG, AMER3, PRSS54, TEX55, SPATA21, and LSM1 proteins. The interaction between ZPBP proteins and other proteins is essential for spermatogenesis and sperm function. The proteins that interact with ZPBP include ACRBP, SSMEM1, SPATA48, TSACC, ARMH4, SPA17, TEX30, ROPN1, and SPACA1. These interactions indicate that ZPBPs have a central role in the protein network involved in zona pellucida binding during fertilization, as well as in different aspects of sperm development and function (Figure 5B).

The SPACA3 protein interacts with several other proteins that are crucial for spermatogenesis and sperm function. These proteins include IZUMO4, ACRBP, SPACA1, IZUMO1, TMEM190, IZUMO2, TSGA10, TCEA2, PRM3, and SPAG11B (Figure 5C). Furthermore, the proteins that interact with CCDC136 in this network are CCDC184, CCDC7, PROCA1, DHRS7B, ALMS1, FAM205A, FAM71B, NT5C1B, ESR2, and CCDC151. The CCDC136 protein as a central node, likely played a significant role in regulating cellular functions and participating in various biochemical pathways and biological processes (Figure 5D).

STRING analysis was conducted on sperm proteins that are related to reproduction (Table 3). The ZPBP protein plays a crucial role in processes like acrosome assembly and spermatid development. In the case of acrosome assembly (GO:0001675), ZPBP is one of three out of twenty proteins involved, with a very low false discovery rate (FDR) of 0.0021, indicating its high significance. ZPBP is also associated with sexual reproduction and acrosomal vesicles. The counts and FDR values further highlight its significant role in relevant networks.

**Table 3:** STRING analysis of sperm proteins related to the reproduction.

Protein (accession)	GO-term	Protein function	Count in network	FD rate	Interaction proteins
ZPBP	GO:0001675	Acrosome assembly (BP)	3 of 20	0.0021	ZPBP, ACRBP, SPACA
	GO:0007286	Spermatid development (BP)	4 of 151	0.0047	ZPBP, ACRBP, ROPN1, SPACA1
	GO:0019953	Sexual reproduction (BP)	5 of 602	0.0140	ZPBP, ACRBP, SPACA1, ROPN1, SPA17
	GO:0001669	Acrosomal vesicle (CC)	3 of 92	0.0223	ZPBP, ACRBP, SPACA1
SPACA3	GO:0001675	Cellular process involved in reproduction (BP)	4 of 317	0.0357	SPACA3, IZUMO1, SPACA1, ACRBP
	GO:0032504	Multicellular organism reproduction (BP)	6 of 575	0.0018	SPACA3, IZUMO1, SPACA1, ACRBP, TSGA10, PRM3
	GO:0022414	Reproductive process (BP)	6 of 1,033	0.0183	SPACA3, IZUMO1, SPACA1, ACRBP, TSGA10, PRM3
	GO: 0019953	Sexual reproduction (BP)	6 of 602	0.0018	SPACA3, IZUMO1, SPACA1, ACRBP, TSGA10, PRM3
	GO:0007283	Spermatogenesis (BP)	4 of 362	0.0498	SPACA1, ACRBP, PRM3, TSGA10
	GO:0002080	Acrosomal membrane (CC)	4 of 21	7.60e-	SPACA3, IZUMO1, SPACA1, MEM190
	GO:0001669	Acrosomal vesicle (CC)	5 of 92	7.60e-	SPACA3, IZUMO1, SPACA1, MEM190, ACRBP
	GO:0002079	Inner acrosomal membrane (CC)	2 of 6	0.0019	SPACA1, MEM190
CCDC136	GO: 0120228	Outer dynein arm docking complex (CC)	2 of 5	0.0084	CCDC151, CCDC114

False discovery (FD); Biological process (BP); Cellular component (CC).

Similarly, SPACA3 protein is involved in various reproductive processes and cellular components. For example, in the context of multicellular organism reproduction (GO:0032504), SPACA3 interacts with proteins such as IZUMO1 and ACRBP, and it is present in 6 out of 575 relevant proteins, with an extremely low FDR of 0.0018, indicating high statistical significance. SPACA3 is also associated with the acrosomal membrane and vesicle, with counts and FDR values showing strong significance, particularly for the acrosomal membrane with an FDR of 7.60e-07. CCDC136 is involved in the outer dynein arm docking complex (GO:0120228), with CCDC136 and its interacting partners constituting 2 out of 5 proteins in this complex, and an FDR of 0.0084. Although the FDR for CCDC136 is slightly higher compared to some other proteins, the results still indicate significance in its structural function within ciliary complexes.

**Figure 6:**
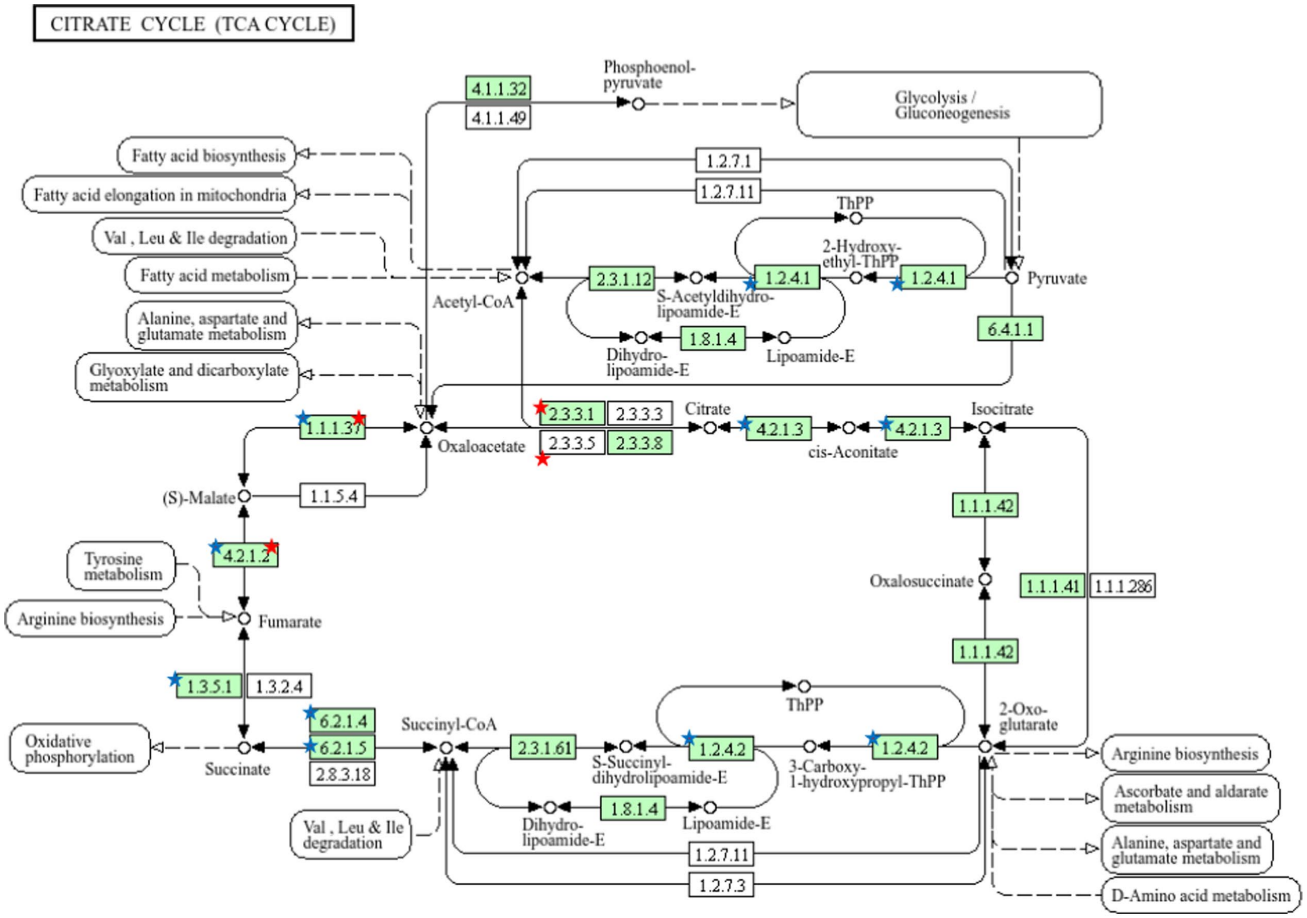
Pathway analysis using the KEGG pathway database identified proteins involved in the TCA cycle. The red star denotes the detected proteins in seminal plasma and the blue star notes the detected proteins in sperm.

The results showed that there are enzymes in the TCA cycle pathway that play an important role in energy metabolism. In sperm, namely Aconitase (4.2.1.3), *α*-Ketoglutarate Dehydrogenase (1.2.4.2), Succinyl-CoA Synthetase (6.2.1.4) and Succinate Dehydrogenase (1.3.51). In seminal plasma, Citrate Synthase (2.3.3.1) while the enzymes found in seminal plasma and sperm are malate dehydrogenase (1.1.1.37) and fumarase (4.2.1.2) (Figure 6).

## 4 Discussion

The characterization of proteomes in ejaculated sperm and seminal plasma is essential for understanding their functional roles in male fertility. In this study, LC–MS/MS was employed to identify the total protein composition of seminal plasma and sperm (Table 1), with findings that are consistent with previous research. Fu et al. (3) identified 864 proteins in buffalo seminal plasma and 2,147 proteins in mature buffalo sperm using LC–MS/MS. Additionally, a study utilizing shotgun proteomics identified 1,695 proteins on the surface of buffalo sperm, with nearly half of these proteins involved in key reproductive processes, including spermatogenesis, sperm maturation, and gamete interaction (17).

The most abundant proteins identified in seminal plasma and sperm in this study play critical roles in motility, energy metabolism, sperm protection, and structural integrity (Table 2). The predominant proteins in sperm TUBB4B, ATP5F1B, CCIN, RAB2A, and GAPDHS are crucial for motility, energy metabolism, and cytoskeletal stability (18–20). Meanwhile, the most abundant proteins in seminal plasma CLU, ALB, HSP90AA1, A2M, and GPI are primarily involved in sperm protection, molecular transport, protein stabilization, energy provision, and enzymatic regulation (21, 22). These findings align with previous studies, which reported that the most abundant seminal plasma proteins (ALB, CLU, and AZGP1) and sperm proteins (TUBB, AKAP4, and ODF2) are associated with enhanced fertility in buffalo bulls (3). In buffalo, sperm quality has been identified as a major factor contributing to male infertility. Previous research has demonstrated that a substantial proportion of reproductive failures can be attributed to poor semen quality (23). These findings highlight the importance of understanding the seminal plasma and sperm proteome as a means to improve fertility outcomes in buffalo breeding programs.

Mass spectrometry techniques, such as LC–MS/MS, facilitate the identification of unique peptides in seminal plasma and sperm, enabling a comprehensive analysis of protein compositions in complex biological fluids. This approach is instrumental in identifying potential biomarkers associated with male fertility. For instance, specific proteins in seminal plasma linked to sperm motility and male infertility have been identified as potential diagnostic markers (24, 25). Gene ontology (GO) annotations of the buffalo sperm and seminal plasma proteomes allowed for the prediction of protein functions, providing valuable insights into key biological processes, including energy metabolism, fertilization, the acrosomal reaction, antioxidant activity, and sperm capacitation (26).

The classification of proteins using PANTHER GO analysis involves several hierarchical stages. Initially, proteins are categorized based on their molecular functions, including catalytic activity, binding affinity, and structural roles. Subsequently, proteins are classified according to their involvement in specific biological processes, such as metabolism, cellular regulation, and reproduction. Lastly, proteins are grouped based on their cellular localization, including the cytoplasm, nucleus, and other reproductive structures (8, 27).

The STRING database serves as a powerful tool for elucidating protein–protein interaction networks, which are essential for understanding the functional roles of proteins in reproductive processes (Figures 5, 6). Among these, ADAM32, a protein found in bull seminal plasma, plays a critical role in sperm quality and function. Proteomic analyses have demonstrated a correlation between its molecular weight and semen quality parameters, including motility, viability, and plasma membrane integrity factors that collectively influence fertility (8, 28). ADAM family proteins, including ADAM32, are implicated in sperm-egg interactions and fertility in both bulls and humans. Additionally, metalloproteinase inhibitor 2, a key regulator of ADAMs, has been linked to bull fertility and sperm DNA integrity (11).

STRING analysis of sperm proteins related to reproduction provides a comprehensive understanding of interaction networks involved in various reproductive mechanisms. One such protein, zona pellucida-binding protein (ZPBP), is crucial for spermatozoa-zona pellucida binding and subsequent fertilization. Notably, ZPBP is more abundant in high-fertility (HF) bulls, making it a potential biomarker for fertility. Proper ZPBP function is essential for sperm-zona pellucida interactions, and its dysfunction can result in compromised fertility (26, 29). ZPBP, with a molecular weight of 36.8 kDa and an isoelectric point (pI) of 9.13, exists in two isoforms: ZPBP1, expressed in spermatids and sperm, and ZPBP2, expressed in the testis. ZPBP1 interacts with ZP2, triggering the acrosome reaction required for fertilization. Disruption of either ZPBP1 or ZPBP2 has been linked to infertility due to abnormal sperm morphology and function (30, 31).

Another crucial protein, SPACA3 (also known as SPRASA or SLLP1), is essential for bovine spermiogenesis and sperm-egg interactions. It plays a significant role in the acrosome reaction and oocyte plasma membrane adhesion (32). SPACA3 interacts with other sperm proteins, including equatorin (EQTN), which facilitates sperm-oocyte fusion through the acrosome reaction (33, 34). Additionally, CCDC136, a testis-specific gene, is critical for acrosome formation and fertilization. Knockout studies in mice have shown that the absence of CCDC136 results in severe acrosome defects, leading to infertility. As a member of the coiled-coil domain-containing (CCDC) protein family, CCDC136 is vital for sperm motility and capacitation. Its importance is further demonstrated *in vitro*, where anti-CCDC136 antibodies significantly inhibit fertilization (35, 36).

Pathway analysis using the Kyoto Encyclopedia of Genes and Genomes (KEGG) database enables the identification of proteins involved in the tricarboxylic acid (TCA) cycle by mapping them to specific metabolic pathways. Kumanresen et al. (29) reported that GO annotations facilitate the characterization of biological processes, cellular components, and molecular functions of differentially expressed proteins. Proteins involved in glycolytic pathways, the mitochondrial respiratory chain, and the TCA cycle are essential for sperm motility and energy metabolism. Recent studies have identified two key enzymes related to the TCA cycle, pyruvate dehydrogenase E1 component subunit alpha-2 (PDHA2) and aconitase 2 (ACO2), which are crucial for ATP production in sperm mitochondria (9).

Proteomic studies on buffalo seminal plasma and sperm have also revealed proteins involved in energy metabolism, including those associated with glycolysis and the pentose phosphate pathway—both of which are functionally linked to the TCA cycle (3, 11, 36, 37). The TCA cycle, also referred to as the citric acid or Krebs cycle, serves as a central metabolic pathway in cellular respiration, generating ATP to meet the high energy demands of sperm cells. This cycle is particularly vital for spermatids, as it fuels sperm motility, which is essential for successful fertilization (38, 39).

Key enzymes such as malate dehydrogenase (EC 1.1.1.37) and fumarase (EC 4.2.1.2) play crucial roles in the TCA cycle. Malate dehydrogenase catalyzes the oxidation–reduction reaction that generates NADH, which subsequently contributes to ATP production via the electron transport chain (40, 41). Given that sperm require substantial energy for motility and fertilization, the TCA cycle serves as one of the primary pathways for ATP synthesis in sperm mitochondria. Additionally, enzymes present in seminal plasma contribute to metabolite availability and redox balance, which are essential for maintaining sperm quality. ATP is particularly critical for microtubule activity, which supports multiple sperm functions, including acrosome formation and activation—both of which are energy-intensive processes (42).

Fumarase is another key enzyme in the TCA cycle, catalyzing the reversible hydration of fumarate to malate, thereby ensuring the continuity of the metabolic pathway (40). In addition to these enzymes, other metabolic components of seminal plasma provide valuable insights into sperm health and function. The findings of this study offer a crucial reference for future research on buffalo sperm function and seminal plasma proteomics, particularly in the context of fertility biomarkers. Moreover, these insights pave the way for the development of molecular tools for bull selection, contributing to the conservation of indigenous Indonesian buffalo breeds and enhancing reproductive efficiency.

## 5 Conclusion

This study presents the first comprehensive proteomic identification of seminal plasma and sperm proteins associated with reproductive functions in the Toraya buffalo. The research underscores the identification of key proteins, such as ZPBP, SPACA3, and CCDC136 in sperm, and ADAM32 in seminal plasma, which are potential biomarkers for fertility. These proteins play significant roles in reproductive processes and their presence could offer new avenues for fertility evaluation and enhancement in buffalo breeding programs.

## Data Availability

The data supporting the conclusions of this research can be obtained from the corresponding author upon a reasonable request.

## Footnotes

^1^ https://www.uniprot.org/proteomes/

^2^ http://pantherdb.org/

^3^ https://david.ncifcrf.gov/home.jsp

^4^ https://version-11-0.string-db.org/
